# Parents’ experiences of initiation of paediatric advance care planning discussions: a qualitative study

**DOI:** 10.1007/s00431-021-04314-6

**Published:** 2021-11-16

**Authors:** Karen Carr, Felicity Hasson, Sonja McIlfatrick, Julia Downing

**Affiliations:** 1 Institute of Nursing and Health Research, School of Nursing, Ulster University, Shore Road , Newtownabbey, BT37 0QB UK; 2 International Children’s Palliative Care Network, Bristol, UK; 3 Makerere University Uganda, Kampala, Uganda

**Keywords:** Advance care planning, Children, Palliative care, Goals of care, Initiation

## Abstract

Advance care planning enables parents to discuss their goal and wishes for the future treatment and care of their life-limited or life-threatened child. Whilst research has identified the barriers clinicians face to initiate such discussions, the views of the parents have received scant attention. This qualitative study, using reflexive thematic analysis, aimed to explore parents’ experience of the initiation of their child’s advance care planning discussions, to help provide an understanding to inform future practice. Single interviews were undertaken with 17 non-bereaved and bereaved parents. Parents reported they had engaged with future thinking but needed time before initiating this with clinicians. They identified the need for a trusted professional and time for private, thorough, non-judgemental discussion without feeling clinicians were ‘giving up’. Parents reported that advance care planning discussions were not always aligned to the dynamics of family life. They felt that health professionals were responsible for initiating advance planning conversations according to the families’ individual requirements. There was an apparent lack of standardised protocols to assist paediatric advance care planning discussion initiation.

*Conclusion*: Initiating advance care planning is a complicated process that needs to be tailored to the specific parent and child situation. Health professionals need to appreciate that parents are key contributors to initiate engagement with advance care planning discussions but that they also require support and care, recognising this may facilitate the building of trust, identified as a key corner stone, of paediatric advance care planning initiation and engagement.

**Table:** 

**What is Known:** *• In paediatric palliative care, parents are challenged with wanting to sustain hope whilst thinking and planning for their child’s end of life. Paediatric advance care planning discussions are often delayed or avoided and triggered by physical deterioration.*	
**What is New:** *• Previous relationship with HCP not essential but parental trust of the HCP is fundamental to Paediatric advance care planning initiation engagement. To build mutual trust health professionals’ approach needs to be professional, respectful and empathetic to the parent and child’s situation.* *• Subtle paediatric advance care planning parent initiation preparation by HCP is often unnoticed by parents but may be the crux of empowering parents to initiate advance care planning conversations themselves as they need to feel a sense of regaining control before planning ahead.*

**Supplementary information:**

The online version contains supplementary material available at 10.1007/s00431-021-04314-6.

## Introduction

Paediatric advance care planning (pACP) is an ongoing conversation that enables the goals, wishes and treatment plans for the child or young person with life-limiting/life-threatening (LL/LT) conditions to be discussed by parents, healthcare professionals (HCP) and, where appropriate, the young person. Such recorded discussions enable all parties to share and clarify information and develop an understanding of the available options [1–3]. The initiation of pACP, however, can be challenging due to unpredictable trajectories and advances in technology which aid survival and life span of the child [4, 5]. Early engagement with pACP is widely advocated [6–10], purported to reduce child suffering, parental anxiety and decisional regret [11–14], enabling HCP to anticipate decisions in potential deterioration scenarios [2, 15, 16].

Unlike the adult setting, pACP is surrounded by a range of legal and ethical issues with regards to a minor’s capacity to consent and the influence of parents and HCP as surrogate decision makers [17]. Despite such differences most research on ACP initiation is adult-centred [18–20], with a dearth of research exploring the concept in children’s settings.

Initiation of pACP is recommended to begin early, ideally close to diagnosis [17, 21–23]; however, research indicates barriers exist. HCP barriers include: lack of training, fears of causing further distress and not knowing the ‘right’ time to initiate [10, 14, 17, 24–28], whilst parents cite lack of time, reluctance to engage, decisional difficulty and lack of trust as barriers [10, 29–32]. The resultant effect is varying levels of involvement in advance care planning discussions [33] and reports from HCP and parents that pACP discussions continue to be commenced later than both parties feel is beneficial [17, 34]. Parent perspective on pACP initiation is absent. This study sought to further examine and understand parents’ experience of the start of their child's advance care planning discussions.

## Method

Qualitative research using semi-structured interviews was adopted as they enabled an in-depth-understanding of parent’s views and experiences of pACP initiation. This research was part of a mixed methods study and has been informed by a previous systematic literature review [16], HCP survey and interviews with HCP. This previous research indicated that the vital aspect of the voice of the parent in pACP initiation was missing and also informed the interview questions and prompts. Given the qualitative nature of the study, the epistemological stance of interpretivism and ontological stance of subjectivity were adopted [35]. COmprehensive consolidated criteria for Reporting Qualitative research (COREQ) were used to structure the study report [36] (Supplementary file 1).

### Sample

A purposeful sample was recruited comprising of parents (age > 18 years) of children (age < 18 years) with LL/LT conditions residing in the United Kingdom (UK) and Republic of Ireland (ROI). Bereaved and non-bereaved parents were eligible (Table 1 inclusion criteria). Recruitment was through several charities to ensure a wide range of diagnosis, age, stage of illness and life expectancy (Supplementary file 2).

**Table 1:** Inclusion criteria

	**Exclusion criteria**
Parents of a life-limited child (diagnosed under 18) who:	Not meeting inclusion criteria
have started discussions on advance care plans	
**or** started decision making about advance plans	
**or** started writing an advance care plan	
are currently over 18 years old,	
feel emotionally and physically able to take part,	
read and speak English,	
are willing to provide written consent to interview,	
live in the United Kingdom (UK) or Republic of Ireland (ROI),	
and, if bereaved, their child died over four months ago	

### Data collection

Parents were invited to participate in an online interview, undertaken between November 2020 and January 2021, with option for dyadic interviews (both parents). All interviews were conducted by KC (female children’s palliative care nurse 20 + years, experienced in qualitative research) and this information was included in the participant information sheet. Parents were asked to share their ‘story’, using a semi structured interview guide (Supplementary file 3) to elicit their experiences of pACP initiation. Interviews were recorded and transcribed verbatim. Field notes were completed to supplement the interview data, assist the interviewer to return to specific issues covered without having to interrupt the interviewee and to aid interviewer with reflection following the interview. Parent and child demographic variables were also collected. Interviews varied in length from one to two hours. Ethical approval was obtained from the Ulster University Research Ethics Committee [REC/20/0029 on 20/5/2020] and LauraLynn Children’s Hospice, ROI [LLREC 05/12/2019 on 12/3/2020]. All participants provided written informed consent.

### Data analysis

Reflexive thematic analysis was used to interpret and make sense of the data as it replicates the values of a qualitative concept. Reflexive thematic analysis focuses on working with the data and reflection on it through six phases of analysis [37, 38] (Supplementary file 4). ‘Themes were generated by gathering groups of coded data with a shared meaning underpinned by a central concept. After codes were initially identified themes were iteratively developed’.

## Results

In total, 17 parents (3 fathers and 14 mothers) were interviewed 3 of whom were couples. One interviewee had experienced the death of two children therefore 15 initiation of pACP discussions were reported upon. The majority were white Christian and had been in a relationship with the child’s other parent at the time of pACP initiation. For parent and child characteristics, see Table 2. Despite different disease trajectories, family structures and locations, there were similar patterns for both bereaved and non-bereaved parents. The resulting reflexive thematic analysis actively generated three main themes regarding parent experience. Quote sources are identified by P plus digit = interviewee identity, D = dad, M = mum and B = bereaved.

**Table 2:** Parent and child characteristics of parents (*n* = 17), ill children (*n* = 15) and families (*n* = 14)

	**Number (*N*)**	**Percentages (%)**
**Parent gender**		
Male	3	18
Female	14	82
**Age parent**		
30–39	6	35
40–49	7	41
> 50	4	24
**Country of residence**		
United Kingdom (England, Wales, Scotland or Northern Ireland)	9	52
Republic of Ireland	8	47
**Work status**		
Full time	11	65
Part time	3	18
Stay at home parent, special leave, sick leave	3	18
**Ethnicity**		
White British	6	35
White Irish	7	41
White other	2	12
Black Caribbean	2	12
**Religious affiliation**		
No religion	6	35
Atheist	1	6
Protestant	1	6
Catholic	4	24
Other Christian	4	24
Other religion or belief system	1	6
**Partnership status at time of pACP initiation**		
Married/cohabiting	17	100
**Current partnership status**		
Married/cohabiting	17	100
**In a relationship with child’s other parent at time of pACP discussion**		
Yes	14	82
No	3	18
**In a relationship with child’s other parent now**		
Yes	14	82
No	3	18
**Highest level of education**		
Secondary school	3	18
Trade/vocational	1	6
Graduate degree	8	47
Post graduate degree	5	29
**Child diagnosis (*n* = 15)**		
Brain tumour	4	27
DMD	2	13
Rare genetic	3	20
Epileptic syndrome	1	7
Genetic progressive neurological	1	7
Batten disease	1	7
Trisomy	2	13
Mucopolysaccharide (MPS) diseases	1	7
**Child age at diagnosis (*n* = 15)**		
Antenatal	2	13
0–11 months	6	40
1–5 years	5	43
6–12	1	7
13–18	1	7
**Age child at initiation discussion (*n* = 15)**		
Antenatal	2	13
0–11 months	3	20
1–5 years	2	13
6–12	4	27
13–18	4	27
**Child gender**		
Female	6	40
Male	9	60
**Child current age**		
Deceased	11	73
1–5 years	1	7
6–12	1	7
13–18	2	13
**Age child died (*n* = 11)**		
0–11 months	3	27
1–5 years	3	27
6–12	1	9
13–18	2	18
≥ 18	2	18
**Time between death and interview (*n* = 11)**		
6–11 months	1*	9
Over 1 year but less than 2	2	18
Over 2 years but less than 3	1	9
Over 3 years but less than 4	2**	18
Over 4 years but less than 5	2	18
Fifth year	3	27
**Other children in family**		
**Yes**	14	100
Other child deceased	1	
Other children under 4 years	7	
One child 5–10 years old	8	
Two children 5–10 years old	1	
One child 11–18 years old	5	
Three children 11–18 years old	3	
One adult child	1	
Two adult children	2	

Percentages may not equal 100 due to rounding

^*^One couple; ^**^Two couples

Prior to their personal experience, some parents were aware of advance planning for adults but were unfamiliar with the concept for children. The term pACP was not commonly recognised but the concept of planning ahead was familiar to all parents.

“There were things in place, but it was never called an advance care plan” (P8MB)

### Theme 1: The route to a place of acceptance

Whether time with their child was brief or long, manifestation of their child’s life-shortening condition and the diagnosis created shock and severe emotional stress. Parents acknowledged this shock impacted on their mental processing ability and their understanding and acceptance of the prognosis.

“If a parent in such a state, perhaps they’re saying, I could be telling you this, and you are receiving it, but it’s going in here, (points to head) and it’s **not** making sense. How do you ask a question when you don’t even understand what you’re being confronted with?” (P12DB)

Parents who accepted they could not change the outcome but could control other aspects were open to pACP whereas those who fought to change the outcome and did not accept the prognosis did not reach a point of accepting pACP initiation. Internal patterns of parental thinking were emotionally complex and embroiled in feelings of guilt of ‘giving up’, combined with anticipatory grieving and the realisation that as a parent they had the responsibility to advocate for their child and be a good parent.

“We got to the stage, where we were the people making the decisive decisions, you know, because we came to the position of that, we were confident that we knew best because we knew (son) best” (P10DB)

Parent’s personality traits such as ‘organiser’ or ‘not a planner’ influenced their participation in pACP discussions with ‘organisers’ accepting or pursuing early pACP initiation attempts.

Avoidance of death talk led parents to reflect that until pACP was initiated, they had no avenue within which their concerns about end of life and death could be discussed.

“… not being able to talk about that end point, even though you think about it, probably from the moment you wake up to the moment till you go to sleep every single day....it needs to be recognised, doesn't it?” (P13M).

For some, spiritual beliefs and religious practices were acknowledged as assisting in parents’ engagement. However, for others, a strong belief in the power to heal opposed acceptance of their child’s death and constrained them from participating in pACP discussions.

“I was confused….the words that were being said to me, did not match up with what the Bible's teaching about. And it's very easy for people of faith to be so focused on living and being protected, protected, that they don't plan or consider death or end of life” (P1MB).

Parents’ engagement with pACP was based on acquisition of knowledge, their level of understanding, and experience and centred on acceptance of the prognosis. To achieve a sense of control and normality, they suppressed emotions, adapted to the child’s situation and become experts on their child’s condition. Nevertheless, embarking on pACP was emotionally challenging; the importance of preserving the child’s life was an ongoing priority, yet this was balanced with witnessing episodes of physical deterioration and continual feelings of loss and grief. Entering pACP was tension driven from the outset. For many the pACP initiation pivot point, following traumatic acknowledgment of inevitability of death, was the need to have clarity for themselves and carers so all could work toward family goals.

“We were of the opinion, and the doctors agreed, it would be no benefit for her, for everybody else. We’d get to keep her around, but it wouldn’t be no benefit for her” (P11DB)

Parents articulated the need to know what to do in an emergency, to know what death may look like, to prepare themselves, their child, siblings and wider family circle for the end of life. Parents identified family and HCP benefits such as clear communication, as the motivation for them to engage in the process with ultimately their child’s best interest, as the main focus (Table 3).

“We do want her treated. We want her looked after. It made me realise that you do you need … to let the hospital know what you want, especially with a child, that, they don't really know us, they don't know her. Mmm…. they [HCP] just see she’s a [genetic condition]and they think ‘well is she here to just pass away? What do they [parents] want us to do?’ And I think it was very, very important that we say ‘no’, she needs proper care” (P17M)

**Table 3:** Parent identified reasons for initiating pACP

Child	
Best interest	“I think if I was to get (son) resuscitated’ I said ‘I’d be doing it for myself, not for him.’ ‘I’d be bringing him back,’ I said ‘to please me because I can’t live without him. Because I feel I can’t live without him. I suppose it just wouldn’t be fair on him. Everything that he’s been through and it’s the right thing for him to do’ if I was like, (son), I said, ‘I don't think I’d like to be living. I don’t think I’d like to be resuscitated’ So I kind of put myself in (sons) shoes to make that decision for him” (P15MB)
Quality of life rather than extending life	“didn’t want to draw out, I had watched them in America having them on ventilators and tube feeding them and no quality of life” (P14MB) “She was tormented nonstop and we didn’t want to be doing anything to prolong that needlessly” (P11DB)
**Parent**	
Questions answered	“For me, there was a sense of needing and wanting to know more and, and wanting to ask questions, but not being given a forum, a forum to do that” (P13M)
Pressure removed as discussions enabled decisions	“If you get it all down in black and white like, you can just put it away then and enjoy the rest of your life rest of his life for him. The memories without having to think about ‘Oh, what’s this and what’s that’. I even, I planned (sons) funeral in advance as well (sons) funerals was planned for five years” (P15MB)
Parent consensus	“She told me this story and I said to my husband, “we need to discuss this” [laughs] I said, because it might just be one of us there and we need to know what the other one thinks about this” (P5M)
Clarity—needing to know	“We wanted to plan the birth, be able to plan the birth. There was risks added to a normal birth, because of the condition. Also, from a psychological point of view, I would not have been able to carry on with the pregnancy without, not knowing what was going to happen” (P8MB)
Parallel planning—knowing the worst scenario plans	“This tumour has got a very high chance of recurring, so I have to have some kind of, in my mind, some kind of preparation for if, if it’s identified that it’s, that it has returned, I need to know what the plan is” (P7M)
Avoid repeating story and plans	“From my understanding of it now, from (son) and what we had done in it, is that instead of having to explain your history, when you go into a&e (Emergency Department) or anything or whatever service you're going to, you’d be able to just hand this over and they can read it and it can be adjusted to whatever needs there are at that time” (P15M)
Discharge process	“It was just, it was just part of the process[discharge] (P10DB)
Avoiding crisis decisions	“…to avoid any more awkwardness where we’d have to be asked or told something we weren’t prepared for, or not in a position to talk about. We didn’t want anything left to chance. Nothing blurry. It was all just there definite” (P11BD)
Clarity in decision making	“difference between, you know, ordinary medical care, we were very clear in our minds and extraordinary care, which may not have been appropriate for a child, like (child) ….. I think the thing about it—is this a bridge to healing? Is this going to help?” (P17M)
**HCP**	
To inform medical staff of required levels of care	“You do you need to sit down and let the hospital know what you want, especially with a child, that they don't really know us, they don’t know her….. Very much seen as helping the medical staff in the event of landing in an A&E (Emergency Department), you know. To sort of move forward with treating her as best they could. And really, as I say, that was mostly our focus, rather than, you know, talking too much about what care we didn't want. It was more, what care did we want” (P17M)
To inform medical staff of limitations of levels of care	“…for the professionals involved, why it was important practically to have it in place” (P13M)
For HCP to be assured discussions have been thorough with parents	“…more about you wanting to re explore it and make sure we're happy with it” (P13M)

### Theme 2: Initiation of pACP as an iterative process

There was an apparent lack of standardised protocols to assist pACP initiation, yet most parents believed it was the HCP responsibility to have the knowledge and skills to start the pACP discussion with them and, when appropriate, following their approval, with their child regardless of the minor’s age.

The trigger for pACP initiation inevitably impacted the process and when, how, who and where it occurred. Triggers could be categorised into two groups: proactive, preparing for an inevitable decline or crisis such as discharge planning requiring a care package with documented decisions required to ensure for HCP clarity, or reactive, in response to decline or crisis, such as relapse, acute physical deterioration or ED attendance where the focus was on attempting to quickly gauge parent expectations and ultimately needing to know limitation of treatment decisions.

“The hospital said he can’t go home without some sort of plan” (P3MB).

“And we’re in it, we were in A and E (Emergency Department). and when they looked at her chart, you know, there was no proper plan there, we hadn’t discussed or thought what would happen in this event, ‘Would she go to intensive care? Would she go to the normal ward?’ or ‘What would happen?’” (P17M)

HCP were reported as adopting multiple individualised subtle or overt pACP initiation approaches (Table 4). For example, parents recognised that some HCP initiated pACP based upon parent readiness cues, whilst others reflected on the subtle ‘drip feeding’ approaches used by HCP to gauge readiness to engage. For example, checking understanding of the severity of the child’s condition and emphasising the need to plan ahead.

“…was more intuitive, I think on her part, and the feeling of understanding what it was, that we needed.” (P13M)

**Table 4:** Initiation approaches by health care professionals

Given blank document to read through
Part of discharge process—to get home this information must be discussed and understood
To ensure certain interventions not commenced e.g., CPR by emergency services when the child at home
An awareness of the legal requirements of paramedics and home support workers to commence CPR if no written documentation indication otherwise
To make it clear to agencies and home care workers of the decisions
To ensure wishes clear regarding not only interventions but life wishes, end of life care and arrangements following death
To avoid repetition of their history or decisions
Parallel planning options

Overt work by HCP to prepare families for discussions such as prewarning of potential decisions ahead, of pACP documentation content or disseminating blank pACP documentation to familiarise parents was seen by most as beneficial.

Whether it was coincidence or parent or professional influence, there were aspects of pACP initiation experienced by parents which affected their experience and made it acceptable or unacceptable. Parents reported that pACP initiation commonly occurred late in the child’s care journey. The manner which it occurred was reported by some as feeling rushed, poorly communicated or lacking in parent preparation with details such as an unwanted absence from their child’s bedside not considered.

“It definitely wasn’t the right time to be starting a conversation that needed time... we were given no choice. A nurse just like bundled us in. So, she (Palliative Consultant) knew she was meeting us that day. She probably had in their diary, but no one said it to us” (P16MB)

When HCP gave of their time in a dignified, unrushed, professional manner parents felt they and their child were valued.

“She just gave us lots of time to ask our questions and at no point was she kind of, I don’t know, she never ever gave us the impression there was a hurry, or she was on a time limit or anything like that” (P5M)

Almost half the parents in the study felt they had initiated or attempted to start a planning conversation; however, not all HCP acted upon parent instigation (Table 5). Parents reported HCP behaviours and verbal and non-verbal language indicating nervousness and discomfort which often resulted in engagement avoidance. Many parents recognised that embarking on such conversations was challenging for HCPs.

“In the end I lost it I said, ‘What are you waiting for?’ Like, this is a fight that I had to do all his life I’m doing again, for him not to be resuscitated. I say ‘he’s getting bad, if anything happens’, I said, ‘and they resuscitate him’ I said ‘because that’s not in his chart’…..I can see it from the professionals point of view, would it be hard, bringing it up with a parent but when the parent is bringing it up with you like that it shouldn’t be as hard as what it was” (P15MB)

**Table 5:** Where initiation discussion took place (*n* = 15)

	**Successful**		**Unsuccessful**	
	**Parent initiation**	**HCP initiation**	**Parent initiation**	**HCP initiation**
Hospital ward	1	1	-	2
Paediatric Intensive Care Unit (PICU)	-	-	-	1
Neonatal Intensive Care Unit (NNICU)	-	1	-	-
Hospital room (in private) (planned whilst child an inpatient)	-	2	-	-
Outpatients department	2	-	1	-
Antenatal appointment	2	-	-	-
Children’s hospice	1	-	-	-
Home	-	1	-	-

Some parents recalled pACP initiation as unpleasant and reported it had left them feeling vulnerable, exposed and unsupported. They felt that some, though experts in their speciality and senior professionals, lacked insight to the parent perspective.

“There was [sic] more people. And none of them introduced and they were standing. So, we were sitting, the doctor was sitting, the other people were standing in a semi-circle just standing watching, saying, not a single word, didn’t say a single word at the end either, nothing. They were just, they were just there” (P16MB)

Initiation of pACP discussions where HCP respected parent expertise and care of their child was recognised, reported positively and valued. Knowing the HCP was not a prerequisite although parents needed to feel an affinity indicating that their child’s best interest was the focus and HCP had the same goals as the family.

“It’s about working at who can have those conversations with, and how they connect with each other professionally…….He was lovely doctor, actually, but not somebody we’ve ever met” (P13M)

None of the families indicated the supportive presence of anyone other than their partner. Almost half pACP discussions commenced without a partner present and were not aligned to the dynamics of family life. The ramifications of lone parent pACP initiation often left one parent informing the other which left no guarantee of information being transferred accurately or indeed at all.

“The difficulty was that daddy and mummy were not communicating very frequently about these things. So, daddy in the first half was with (Child’s name) in the hospital. And he was being informed with all these things, he was filled with all this information. But it's again very difficult to come home and tell mummy right we need start planning end of (Child’s name) life. How do you even begin that kind of conversation? What words do you use? Do you do it after lunch? Do you do after the kids are in bed? Do you go out for a meal (laughs)? So, it’s very impossible to fit into everyday life” (P1MB)

### Theme 3: Becoming a team—parent and HCP trust

Trust was at the centre of the pACP initiation process for parents and viewed as a prerequisite to facilitate their engagement. Parents needed, not only to trust HCP but also, to feel that HCP trusted them. Trust was aligned to HCPs recognising the parent’s role and acting in the child’s interest not perceived as ‘giving up’, ‘limiting’ or ‘overpowering’ parent’s wishes for their child. Parents who felt they were encouraged to ask questions and trusted HCP would not be dismissal or judgemental appeared to acquire a greater understanding of the journey ahead and initiated or accepted pACP discussions.

“Whenever we had questions, maybe more visceral questions about things in particular that you might not feel comfortable asking the doctor, or somebody that you might be close to, but not that close, we would always ask her” (P8MB)

When HCP validated and empathised with parents and connected with the child, whether planned or impromptu, parent or HCP led pACP initiation occurred.

“We trusted (sons) team and (sons) consultants at that stage, you know. And if they were, if this is what they believed was in (Son’s) best interests, well, then we believed them that they had (Son’s) best interests at heart” (P10DB)

Previous poor experiences, inadequate communication and perceptions of contradictory goals led to feelings of distrust. Mistrust led to scepticism and resulted in parents believing that HCPs were focused on the condition, not the child and suspicion that pACP was being introduced to limit treatment. These perceptions resulted in parents disengaging when attempts were made by HCP to initiate.

“Then you say, look, I need to ask other doctors, I need a second opinion. And they take it as a as an insult and is that you don’t even trust them for this…. then this lack of trust builds up…. then you, you, you confront them because you don’t have trust” (P4MB)

Parents who reported actual or potential, poor or dangerous care such as medication errors felt HCP treated these as unwarranted challenges rather than appropriate interjection. In situations where parents confronted HCP, they worried they were labelled as a ‘difficult’ which had negative consequence for pACP initiation.

“….he (child’s dad) already feels like he’s already on the backfoot… he really struggles to feel like his, his questions and queries are warranted, and that they’re taken seriously… he was perceived as being aggressive because he challenged a doctor, an on-call doctor about (sons) condition” (P7M)

Without trust, parents avoided participating in pACP.

## Discussion

ACP for children has been recognised as a process from initiation to documentation completion [15] but this study identifies that there is a preinitiation process required to build foundations. Parental acceptance of their child’s condition and a trusting relationship with a capable HCP were vital prior to initiation. Trusting relationships have been identified in previous research as necessary for parents to participate in planning discussions [15, 22, 23, 30, 33, 39–42]. Generally developing trust is accepted as a gradual process which is better achieved during times of low stress and when the child is stable [43]; however, this study found that parents did not necessarily need to know HCP for a prolonged period to develop the trust and therapeutic alliance required for pACP initiation. Referred to as the third level of trust [44], pACP initiation went beyond entrusting the HCP with the care of the child to a higher level where parents needed to feel a sense of safety to expose their inner fears and thoughts. This was based on parent perceptions that the HCP would listen, ‘cope’ and respond knowledgeably and in a non-judgmental manner.

The initiation of pACP did not occur in isolation, rather it is was shrouded in complex processes where the parent must care for the ill child, balance home and family life whilst being acutely aware and accept future loss [45, 46]. Similar to previous research [47], some parents recovered from the shock of prognosis to act as advocates and protectors, ready to engage in conversations that placed the care of their child centre. However, for others, the lack of acceptance and the need to retain hope and faith led to parental delays confirming earlier research [29, 30, 48].

As seen in previous research [21, 49], results demonstrated that triggers for pACP initiation varied for parents and HCP, with physical deterioration the key trigger [3, 22, 49]. Informing parents of the pACP concept unrelated to their child was not recognised as having occurred or did not occur.

This study found that parents, who accepted their child’s prognosis, attempted to actively initiate pACP or accepted conversations with HCPs knowledgeable in their child’s condition. However, it is difficult to gauge if parents initiated discussions following subtle preparation by HCP, or if it was proactive parent pACP initiation. Findings from this study suggest an aspect of subtle parent preparation by HCP often unnoticed by parents. Although many parents in this study felt they themselves had initiated pACP, they believed HCP held the responsibility to initiate pACP discussions, which reflected results from other studies [15, 39, 50].

In crisis situations, pACP conversations focused on medicalised decisions sometimes occurring with a lone parent being present, such as withholding treatment or resuscitation status, resulting in many parents equating pACP with end-of-life decisions [51]. Consequently, holding emotive conversations in response to crisis situations led parents to expressing emotive responses and indications of distrust.

Practice could be improved if HCP informed parents about pACP verbally or through parent information leaflets before it is personally required. If HCP commenced pACP discussions with no preconceived goals apart from educating parents about pACP and informing parents that they can start to think of pACP anytime, then pACP initiation would be easier. Early pACP initiation attempts can assist in identifying disagreement and enable more time to reach agreement. Past grievances must be acknowledged as there is no moving forward unless a trusting relationship formed and maintained. In complex cases where expert pACP initiation is required, then early referral to knowledgeable HCP is essential.

Understanding that there are vital processes prior to pACP initiation is important in the planning of policies and protocols. Further research to gauge a deeper understanding on how parental HCP trust develops would potentially assist with initiation.

The adoption of different recruitment routes enabled access to a wider diversity of parents of children with differing conditions resulting in a variety in pACP initiation journeys and parents from differing backgrounds and experiences. The inclusion of both active and bereaved parents in this study enabled experiences to be reported from a retrospective and current stance, heightening understanding of this complex process. However, the reliance on retrospective views may have introduced memory recall basis questioning the generalisability of the findings. Nevertheless, active parents reported similar experiences to bereaved parents across the study. Similar to other studies, there is an over-representation of third-level educated parents whose views and experience may not be representative of parents from differing backgrounds [32, 52]. Parent participation in pACP is nuanced, complex and required the capable, motivated HCP with opportunity.

## Conclusion

This study outlines parent experiences of pACP initiation. Consideration that pACP is surrounded by the anguish of parents who themselves require support and who need time to accept and engage may facilitate the building of trust, identified as a key corner stone of pACP initiation and engagement. Throughout this process, parents adapt and transition their thinking and roles in an attempt to cope, remain in control and be a good parent. HCPs need to recognise this transitional journey to be able to respond to subtle or overt parental cues and be prepared for pACP initiation opportunities. A challenge for HCP is to value parents’ expertise and help to navigate parents through transition to a mutual trust where advance planning and eventually end of life discussions can occur, described as ‘the delicate dance of figuring it out’ [53].

## Supplementary information

Below is the link to the electronic supplementary material.

Supplementary file1 (DOCX 25 KB)

Supplementary file2 (DOCX 15 KB)

Supplementary file3 (DOCX 19 KB)

Supplementary file4 (DOCX 14 KB)

## Data Availability

NVivo Transcriptions are available on request.

## Abbreviations

ACP: Advance care planning
HCP: Healthcare professional
LL/LT: Life-limiting disease/life-threatening disease
pACP: Paediatric advance care planning
ROI: Republic of Ireland
UK: United Kingdom
