# Supplementary material for: Parents’ experiences of initiation of paediatric advance care planning discussions: a qualitative study
Source: Eur J Pediatr. 2021 Nov 16;181(3):1185–96. doi: 10.1007/s00431-021-04314-6 (PMC8897342; doi:10.1007/s00431-021-04314-6)
Supplement: Supplementary file 2 — Supplementary file2 (DOCX 15 KB) [file 431_2021_4314_MOESM2_ESM.docx]

| **Charities used for recruitment** | |
| --- | --- |
| Together for Short Lives | UK’s leading registered charity (England and Wales :1144022 and Scotland: SC044139) for children living with life-limiting conditions and their families. |
| SOFT UK, | Registered Charity: 1002918 (England and Wales) SC043341 (Scotland), is the UK support organisation for Trisomy 13 and 18. |
| Duchenne UK and | Registered Charity (No. 1147094) which fund and accelerate treatments and a cure for Duchenne Muscular Dystrophy (DMD). |
| Batten Disease Family Association (BDFA). | Batten Disease Family Association (BDFA) is a registered UK national charity (England & Wales 1084908 / Scotland SC047408) which aims to support families and raise awareness and facilitate research into this neurodegenerative disease. |
| The Daniella Logun Foundation | Registered Charity Number: 1189746. https://www.facebook.com/TheDaniellaLogunFDN |

**Supplementary file 2**

**Recruitment method** - A variety of methods were used by the charities including direct recruitment by charity support staff or an identified gatekeeper, website posters, posters on the group’s private social media pages or information shared with individuals signed up with the charities to participate in research.
