# Supplementary material for: Parents’ experiences of initiation of paediatric advance care planning discussions: a qualitative study
Source: Eur J Pediatr. 2021 Nov 16;181(3):1185–96. doi: 10.1007/s00431-021-04314-6 (PMC8897342; doi:10.1007/s00431-021-04314-6)
Supplement: Supplementary file 3 — Supplementary file3 (DOCX 19 KB) [file 431_2021_4314_MOESM3_ESM.docx]

**Supplementary file 3**

**INTERVIEW SCHEDULE**

**Study title:** **Exploring parents experience and perspectives on the initiation of paediatric advance care planning**

**Warm-up**

The researcher ensures that the participant feels comfortable and ready to start the discussion and thanks participant/s.

**Opening Statement**

- Review of purpose, rules and confirm consent forms have been signed and understood.
- Remind participants that they can withdraw at any stage
- Disseminate demographic questionnaire (5minutes)

The participant will be reminded of the aim of this study and the researcher will clarify the terms of confidentiality regarding this study as follows: “*Exploring parents experience and perspectives of the initiation of paediatric advance care planning”* You are asked not to identify any health professional in your responses. As per the PIS and Consent form all disclosed information will be treated confidentiality unless required by law i.e. there is a risk to yourself or others.

- Gain verbal consent on mode of recording (video and/or digital voice recording). If permission is not given field notes will be made.
- General chat building of rapport to enable appropriate reduction of discomfort regarding interview and sensitive topic.

|  | **Questions and suggested Probes**  *(depending on answers to initial questions not all questions may need asked).* |
| --- | --- |
| **1** | **Please can you start by telling me about <child’s name> and your family?***Probe:*  *When were you aware that (child) was unwell?*  *When was (child) diagnosed?*  *Where were they at this time—home, hospital, PICU/NNICU/ ward* |
| **2** | **What knowledge did you have about ACP before it was started for <name>?**  *Probes:*  *Heard anything from other parents, newspapers, TV, social media, professionals?* |
| **3** | **Please tell me about your experience of the start of the pACP conversation/ discussion for your child (Name).**  *Probes:*  *Who started the discussion?*  *What was said?*  *Where did it take place?*  *When?*  *Why?* |
| **4** | **Recalling your thoughts of when pACP/ planning ahead first mentioned? How did you feel?**  *Probe:*  *What was most difficult or uncomfortable?*  *How did your feelings (mood, fatigue) affect you initiating/ agreeing to start pACP or not? Did you have any worries/ fears when pACP first mentioned? Were you relieved?*  *Did you have concerns about how others would react if they knew you had a pACP?* |
| **5** | **Had there been previous attempts by HCP/ you to get the pACP discussion started?**  *Probe:*  If yes – please tell me about them. Why didn’t it work? Do you think it was too soon to start this discussion?  If no, do you think you would have liked to have been offered the opportunity to start discussions earlier?  Were there discussions within the family/ with friends about <childs name> and future and potential decision making? |
| **6** | **What factors influenced/motivated your decision to have pACP/ discussion?**  *Probe: Child’s quality of life? Prognosis? Child asked?*  *Probe: What was the role/influence of other immediate family members on the pACP,* |
| 7 | **Were you aware of/ did you use any parent support ACP resources?**  *Probe: Info online, from professionals, from other parents, leaflets? How did the content meet your needs? How could it be improved?* *Did you see the pACP documentation before using it?* |
| 8 | **Did your child have any involvement in the initial/ eventual discussion?**  *Probe: Please explain* |
| 9 | **What factors may have/ did support you initiate pACP?**  *Probe: Other families experience. Wanting to do the right thing for son/ daughter. Seeing the documentation. Wanting your/ your child’s wishes followed.* |
| 10 | What do you think would help increase the number of pACP discussions being started and increase parent and HCP confidence in initiating pACP? |

**Warm down/ Debrief exercise**

Reflect on main issues discussed.

Ensure participant composed and aware of support and information services available.

Advise participant to expect a supportive follow up telephone call within 24 hours.

**Final thank you.**
