# Supplementary material for: Parents’ experiences of initiation of paediatric advance care planning discussions: a qualitative study
Source: Eur J Pediatr. 2021 Nov 16;181(3):1185–96. doi: 10.1007/s00431-021-04314-6 (PMC8897342; doi:10.1007/s00431-021-04314-6)
Supplement: Supplementary file 4 — Supplementary file4 (DOCX 14 KB) [file 431_2021_4314_MOESM4_ESM.docx]

| **Supplementary file 4**  **Reflexive thematic analysis phases** | |
| --- | --- |
| **Phase** | **Description of process** |
| 1 | Two researchers (KC,FH), were involved. Both researchers (re)read the transcripts to become familiar with the data (KC,FH) |
| 2 | Provisional ideas were independently noted, and each researcher coded and described the text with meanings, both overt and implicit. NVivo 12 was used by one researcher (KC) to classify, arrange and sort. |
| 3 | Themes were developed based on codes, analytical memos, and feedback from research team discussions |
| 4 | Selective illustrative quotes were discussed with the wider research team (SMcI, JD) with themes continuously developed into meaningful patterns |
| 5 | Patterns were discussed in team meetings and feedback led to their refinement |
| 6 | Production of the study report |
